# Gold Leaf

**Published:** 1885-02

**Authors:** 


### Gold Leaf.

If a sheet of gold leaf is held up against the light, it appears to be of a vivid dark green color; this means that the light is transmitted through the leaf. When it is considered that this leaf is a piece of solid metal, a better idea of the extreme tenuity of the leaf can be comprehended than by any comparison by figures; nothing made by the hand of man equals it in thinness. This extreme thinness is produced by patient hammering, the hammers weighing from seven to twenty pounds, the lighter hammers being first used. When the true method of of this beating is understood, the wonder expressed sometimes that gold leaf beating should not be relegated to machinery ceases; the art belongs to the highest department of human skill and judgment. Apprentices have served a term, and have been compelled to abandon the business, because they never could acquire the requisite skill and judgment combined necessary to become successful workmen.

The only pure gold leaf is that used by dentists for filling carious teeth, and it is called foil. It is left much thicker than the gold leaf for gilding—indeed, it could not be beaten so thin; for thin, or leaf gold, an alloy of silver and copper is required to impart the requisite tenacity. Dentist's foil weighs six grains, five, four, and three grains per sheet, or leaf, according to its thickness. The last operation on the leaf is annealing. This is done over a charcoal fire, the leaf being laid singly in a sort of corn popper—a square receptacle with wire bottom at the end of a handle—over which is held a similar cover, to prevent the flame from carrying the leaf away. An instant's exposure to the flame induces a red heat, when the leaf is laid on a sheet of a book.

The material for gold leaf and dentist's foil is coin gold. The gold is precipitated by muriatic and nitric acids, over a fire to separate the gold and silver, the copper of the alloy passing off in the heat. The silver from gold coin amounts to about seven pennyweights to $800 worth of coin—the amount usually treated at a time. This reduction and separation of the metals is the usual method, and does not require special description.

The pure gold is then melted in sand crucibles with the proper proportions of silver and copper to produce the color of leaf desired, very fine ornamental effects being produced in gilding with leaf of differing shades. The fluid metal is poured into iron moulds, making bars seven inches long, one and an eighth inches wide, and one-fourth of an inch thick. These bars are forged, like iron, between anvil and hammer, to even the edges, and then rolled in powerfully geared rolls, to a ribbon not thicker than writing paper and one inch wide. Of course, in the rolling, as in all the processes, there must be occasional annealings.

Now comes the first of the beating processes. These squares of gold (one inch square) are placed in a pile alternating with larger squares (four inches or more) of "kutch" paper, a material made from a pulp of animal membrane—raw hide, intestines, etc.—and the outside of the pile receives a square of parchment. The hammering then begins with a seven pound hammer on a block of marble that rests on a solid foundation. After one hour's beating, the pile is warmed at a fire to anneal the gold, a process requiring care, so that the kutch paper be not burned. Four hours of beating suffices for this preliminary process, 180 squares of gold being treated in one pile. The final process requires great skill. The partially beaten squares are packed as before, but with alternates of gold-beater's skin, until the pile contains 900 sheets. The beating is continued with increasingly heavier hammers, until the final finish with the twenty pound hammer. The gold-beater's skin comes from England, and the best of it—and the most of it—is made by one family—Frederick Perkins'. The skin is so thin as to be almost transparent, and yet it is double, two thicknesses. It is prepared from the larger intestine of the ox. Each sheet of the skin is rubbed on each side, before the pack is made and whenever the pack is rearranged (placing the outer gold in the center and *vice versa*), with a powder made from calcined gypsum of a very pure sort, imported from Germany. This is to prevent the gold from sticking to the skin.

In beating, the work of spreading the gold is from the center of each square of gold out toward the edges, and the finished squares are thicker at the edges than in the center. A contrary spreading would split the edges and ruin the squares. In rearranging the squares in the process of beating they are sometimes torn, but another piece laid on as a patch, lapping over the torn place, will be firmly welded in the after beating.

The finished squares are cut to a size of three and three-eighths inches, and packed in a "book" holding twenty-five sheets, the paper leaves being rubbed with red ocher to prevent sticking. These books of twenty-five sheets are sold at from thirty to forty cents each. The cutting of the leaf is done by knives, which are simply sligs of the outer shiny shell or skin of the Malacca cane such as is used for walking sticks. The outer rind contains silex or flint in minute, invisible particles, forming a peculiar edge. Steel will not answer the purpose.—*Scientific American.*

